# Supplementary material for: Plumbagin Suppresses Breast Cancer Progression by Downregulating HIF-1α Expression via a PI3K/Akt/mTOR Independent Pathway under Hypoxic Condition
Source: Molecules. 2022 Sep 5;27(17):5716. doi: 10.3390/molecules27175716 (PMC9457614; doi:10.3390/molecules27175716)
Supplement: Supplementary file 1 [file molecules-27-05716-s001.zip › molecules-1848372-supplementary.pdf]

Supplementary Material

Plumbagin suppresses breast cancer progression by down-regulating HIF-1α expression via a PI3K/Akt/mTOR independent pathway under hypoxia condition

Supawan Jampasri [1], Somrudee Reabroi [1], Duangjai Tungmunnithum [2], Warisara Parichatikanond [3,4] and Darawan Pinthong [1,*]

[1] Department of Pharmacology, Faculty of Science, Mahidol University, Bangkok 10400, Thailand

[2] Department of Pharmaceutical Botany, Faculty of Pharmacy, Mahidol University, Bangkok 10400, Thailand

[3] Department of Pharmacology, Faculty of Pharmacy, Mahidol University, Bangkok 10400, Thailand

[4] Center of Biopharmaceutical Science for Healthy Ageing (BSHA), Faculty of Pharmacy, Mahidol University, Bangkok, 10400, Thailand

* Correspondence: darawan.pin@mahidol.edu

Table S1. Lists of primer sequences used for qRT-PCR

Gene	Sequences (5′-3′)	
	**Forward**	**Reverse**
HIF-1α	TTTTGGCAGCAACGACACAG	TTTTCGTTGGGTGAGGGGAG
VEGF	ACAACAAATGTGAATGCAGACCA	TACCGGGATTTCTTGCGCTT
VEGFR-2	TGTGTATGTCCCACCCCAGA	GGGAGGAATGGCATAGACCG
GAPDH	GACAGTCAGCCGCATCTTCT	ACCAAATCCGTTGACTCCGA

Figure S1. Full-length blots representing the protein expression of HIF-1α and β-actin as shown in Figure 4A of the manuscript.

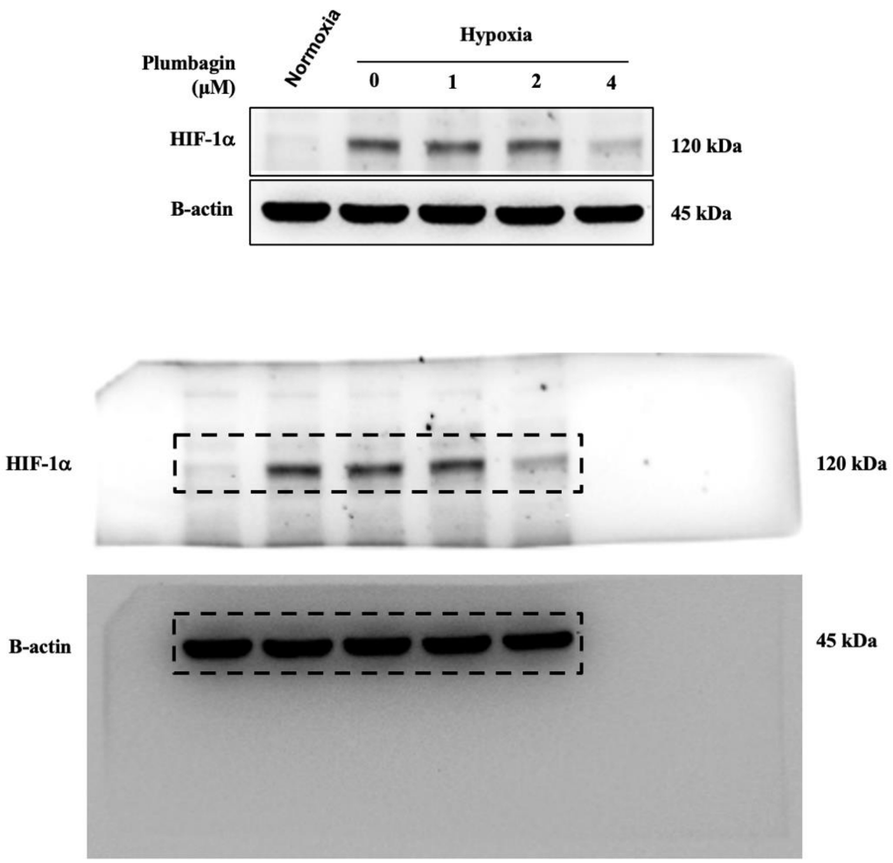

Figure S2. Full-length blots representing the protein expression of VEGF and β-actin as shown in Figure 5C of the manuscript.

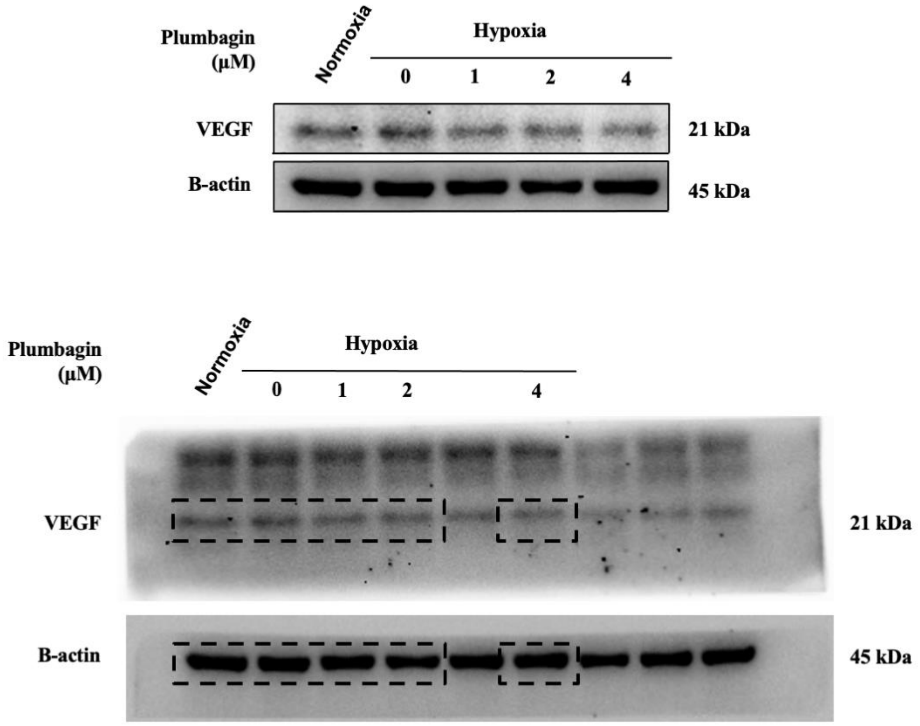

Figure S3. Full-length blots representing the protein expression of PI3K/ Akt/ mTOR/ HIF-1α, and β-actin as shown in Figure 6A of the manuscript.

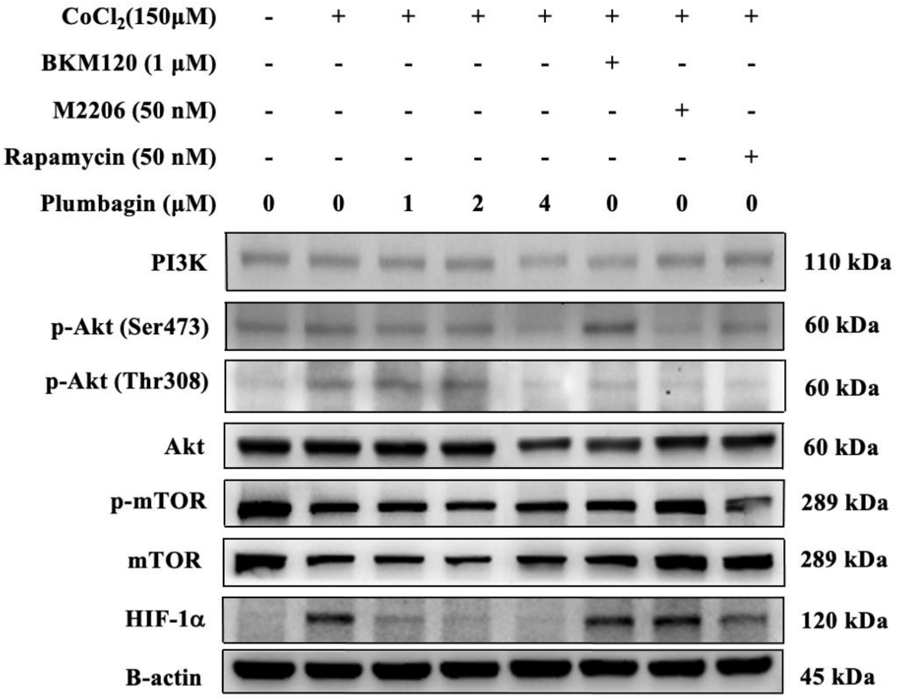

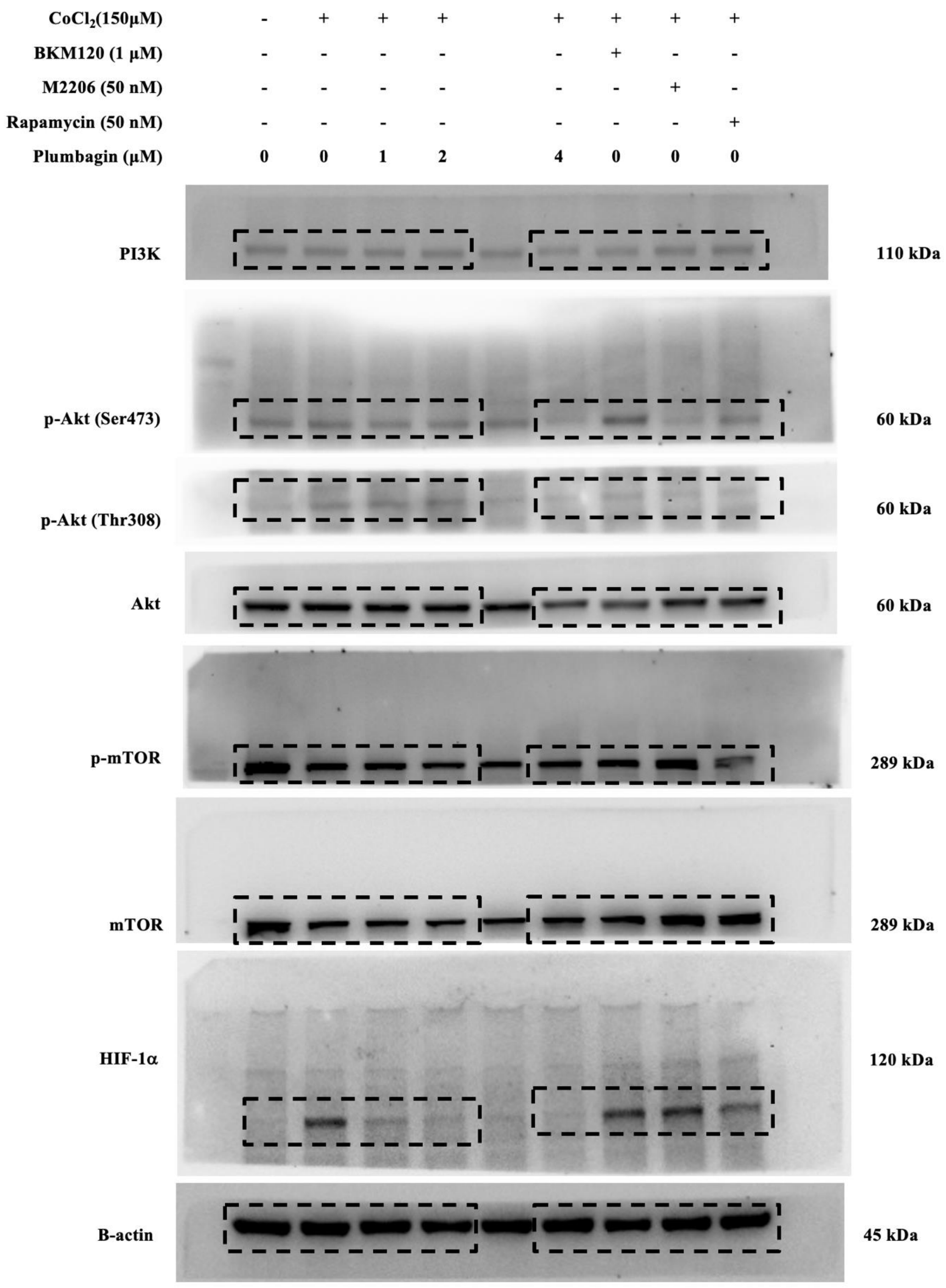

Remark: The membranes were cut separately, according to size of protein marker, in order to incubated with specific 1°Ab, 2°Ab and 5% nonfat dry milk in TBS-T, respectively before detected and imaged the bands with the ChemiDocTMTouch Imaging system.
